# Codon Optimization, Expression in *Escherichia coli*, and Immunogenicity of Recombinant Chinese Sacbrood Virus (CSBV) Structural Proteins VP1, VP2, and VP3

**DOI:** 10.1371/journal.pone.0128486

**Published:** 2015-06-12

**Authors:** Dongliang Fei, Haochun Zhang, Qingyun Diao, Lili Jiang, Qiang Wang, Yi Zhong, Zhaobin Fan, Mingxiao Ma

**Affiliations:** 1 Animal Husbandry and Veterinary Institute, Liaoning Medical University, Jinzhou, China; 2 Honeybee Research Institute, the Chinese Academy of Agricultural Sciences, Beijing, China; 3 Liaoning Water Conservancy Vocational College, Shenyang, China; Centro de Biología Molecular Severo Ochoa (CSIC-UAM), SPAIN

## Abstract

Chinese sacbrood virus (CSBV) is a small RNA virus family belonging to the genus *Iflavirus* that causes larval death, and even the collapse of entire bee colonies. The virus particle is spherical, non-enveloped, and its viral capsid is composed of four proteins, although the functions of the structural proteins are unclear. In this study, we used codon recoding to express the recombinant proteins VP1, VP2, and VP3 in *Escherichia coli*. SDS-PAGE analysis and Western blotting revealed that the target genes were expressed at high levels. Mice were then immunized with the purified, recombinant proteins, and antibody levels and lymphocyte proliferation were analyzed by ELISA and the MTT assay, respectively. The results show that the recombinant proteins induced high antibody levels and promoted lymphocyte proliferation. Polyclonal antibodies directed against these proteins will aid future studies of the molecular pathogenesis of CSBV.

## Introduction

Chinese sacbrood disease (CSD) is a highly contagious and lethal infection that affects the larvae of the Asian honeybee (*Apis cerana*). CSD is characterized by rapid onset and spread [1,2]. Viral outbreaks result in larval deaths in colonies, and if the resistance of the adult bees decreases, the entire colony may collapse [3,4]. CSD was first described in Guangdong, China, in 1972, and subsequently spread throughout China and the countries of Southeast Asia, causing lethal disease in individual bees, or the collapse of entire colonies [5]. In 2008, a CSD outbreak in Liaoning Province, China, spread to nearly 30,000 honeybee colonies, accounting for as much as 75% of all colonies, thereby causing great losses in Chinese honeybee produce [6].

The causative agent of CSD is the Chinese sacbrood virus (CSBV) [7]. CSBV, a member of the picornavirus family, is an icosahedral, non-enveloped virus, 26–30 nm in diameter, and it belongs to the genus *Iflavirus* [8,9]. It is a single-stranded, positive-sense RNA virus with a genomic sequence composed of about 8,800 nucleotides [7]. Its genome contains a main open reading frame, encoding a polypeptide comprising four structural proteins (VP1, VP2, VP3, and VP4). Three structural proteins have been reported in CSBV, although the VP4 protein has not been found [7,10,11]. At present, structural protein studies of CSBV have focused on genome sequencing and analysis [12,13], and the functions of the proteins have not been reported. In our previous studies, we attempted to express the structural proteins VP1, VP2, and VP3 in the pGEX prokaryotic expression system, although the result of the protein expression was unsatisfactory, which adversely affected further research. To improve the production of recombinant VP1, VP2, and VP3 proteins, we designed and synthesized recoding structural protein genes in accordance with the codon preference characteristics of the *Escherichia coli* expression system, without changing the amino acid sequences sequences of the wild-type proteins (roVP1, roVP2 and roVP3). Following their expression and purification, we studied the immunogenicity of the recombinant proteins. This research lays the foundation for revealing the molecular pathogenesis of CSBV infections and the development of a polyclonal antibody against the virus.

## Materials and Methods

### Ethics Statement

All animal experiment were conducted under protocols by the Ethics Committee and the Experimental Animal Center of Liaoning Medical University, and was performed in accordance with local ethical guidelines.

### Virus, Strains, Plasmids, and Main Reagents

CSBV was isolated, identified, purified, and preserved by the authors’ laboratory [7], and its genome has been sequenced (Genbank accession no. HM237361.1). *E*. *coli* strains DH5α and BL21(DE3) were purchased from TransGen Biotech (Beijing, China); the expression vector pGEX-6P-1 was from Invitrogen (California, USA); PCR premix, restriction enzymes, AMV reverse transcriptase XL, and DNA Ligation Kit were obtained from TaKaRa (Dalian, China); the SV Total RNA Isolation System, horseradish peroxidase (HRP)-labeled goat anti-rabbit IgG were from Promega (Wisconsin, USA); the GST-Tagged Protein Purification Kit was from Clontech (New Jersey, USA); the BCA protein assay kit was from Sigma-Aldrich (Wisconsin, USA); GST(91G1) rabbit mAb was from Cell Signaling Technology (Boston, USA); rabbit anti-CSBV IgG was producted and stored by the authors’ laboratory; and HRP-conjugated rabbit anti-mice IgG was obtained from Abcam (London, UK).

### Construction of Wild Type Gene Expression Vectors

Total viral genomic RNA was extracted using the SV Total RNA Isolation System according to the manufacturer’s instructions. Then, cDNA was synthesized using 10 μL of total RNA, AMV reverse transcriptase XL, and random oligo(dT)18 as primer. Three pairs of primers for *VP1*, *VP2*, and *VP3* genes amplification were designed based on the CSBV mRNA sequence (Genbank accession no. HM237361.1). At the same time, restriction enzyme sites were inserted (Table 1). PCR reaction mixtures (25 μL) contained 2.5 μL of cDNA, 0.2 μM of each primer, and 15 μL of PCR premix. PCRs were conducted as follows: denaturation at 94°C for 4 min, followed by 30 cycles of 94°C for 45 s, 55°C for 45 s (*VP1*), or 60°C for 45s (*VP2*), or 58°C for 45 s (*VP3*), 72°C for 60 s, and a final extension of 72°C for 10 min. The PCR products were purified, double-digested, extracted, and inserted into the restriction enzyme sites of the pGEX-6P-1 vector. The expression pGEX-6p-VP1, pGEX-6p-VP2, and pGEX-6p-VP3 vectors were constructed and identified by gel electrophoresis after double-digestion and DNA sequencing (Dalian Takara Biotechnology).

**Table 1:** Synthetic oligonucleotides for amplification of *VP1*, *VP2*, and *VP3* genes.

Target gene	Primers	Sequence (5′ to 3′)	Restriction enzyme sites
*VP1*	VP1 F	5′-GCGGATCCGATAAACCGAAGGATATAAG-3′	*Bam*HI
	VP1 R	5′-GCCTCGAGTTATTGTACGCGCGGTAAATA-3′	*Xho*I
*VP2*	VP2 F	5′-GCGGATCCCAGGGGGACGCTACACAG-3′	*Bam*HI
	VP3 R	5′-GCCTCGAGTTACTGGGTCAGTGGAACTT-3′	*Xho*I
*VP3*	VP3 F	5′-GCGAATTCGACACTGGTGCTAAAG-3′	*Eco*RI
	VP3 R	5′-GCCTCGAGTTACTGTTGTGCTGCTCCCG-3′	*Xho*I
	VP3 R	5′-GCCTCGAGTTACTGTTGTGCTGCTCCCG-3′	*Xho*I

### Construction of Recoding Gene Expression Vectors

To improve the expression levels of the *VP1*, *VP2*, and *VP3* genes, the gene sequences were recoded on the basis of the codon preference characteristics of a prokaryotic expression system, without changing the amino acid sequence of the corresponding proteins [14,15]. The strategy of codon optimization developed in this study is known as ‘one amino acid-one codon’, in which the most preferred codon of the host for a given amino acid is used in the target sequence [16]. Online optimization software (http://www.jcat.de/ and http://genomes.urv.es/OPTIMIZER/) were utilized for codon design. The recoding genes were synthesized by Dalian Takara Biotechnology (*oVP1*, *oVP2* and *oVP3*). For convenience, the restriction sites *Bam*HI/*Xho*I, *Bam*HI/*Xho*I, and *Eco*RI/*Xho*I were inserted into the *oVP1*, *oVP2*, and *oVP3* genes, respectively, and the vectors containing synthesized coding regions were named pMD-18T-oVP1, pMD-18T-oVP2, and pMD-18T-oVP3, respectively. These vectors were then double-digested and inserted into the corresponding restriction enzyme sites of the pGEX-6P-1 vector, and the expression pGEX-6p-oVP1, pGEX-6p-oVP2, and pGEX-6p-oVP3 vectors were constructed and identified by double-digestion and sequencing.

### Inducible Expression and Purification of Recombinant Protein

The *E*. *coli* BL21(DE3) strain was transformed with the identified plasmids. Six single bacterial colonies were selected from the transformants. The selected single bacterial colonies were inoculated into 5 mL of Luria-Bertani(LB) medium and incubated at 37°C for 10 h. The cultures (1000 μL) were inoculated to 100 mL LB (100 μg/mL Amp) and cultured at 37°C until the absorbance at 600 nm reached 0.6, then isopropyl β-D-1-thiogalactopyranoside (IPTG) was added to induce protein expression. To increase the expression level, inducible conditions were optimized, including the concentrations of IPTG, temperatures, and induction durations. At the same time, the uninduced and vector control groups were established in parallel. After induction, cells were harvested by centrifugation at 4000 rpm for 10 min, then the supernatants discarded, and the pelleted bacteria of roVP1, 2, and 3 were weighed, respectively. The pellets were resuspended using 5 mL PBS buffer, then were lysed using twenty 10-s rounds of ultrasonication on ice (Ultrasonication power: 40W; Probe type: Ф3mm). After ultrasonic disruption, lysates were centrifuged at 8000 rpm and 4°C for 15 min, and the supernatants (soluble fraction) carefully transferred to a clean tube, then the proteins of roVP1, 2, and 3 were purified using the GST-Tagged Protein Purification Kit. The concentration of purified roVP1, 2, and 3 were determined using the BCA kit, then the overall yields of roVP1, 2, and 3 proteins were assessed by calculating the purified protein content per gram of pelleted bacteria, respectively. At the same time, the supernatants and the purified samples were analyzed by SDS-PAGE.

### Western Blot Analysis

Purified fusion proteins were separated by 15% SDS-PAGE gels and transferred onto a nitrocellulose membrane according to the manufacturer’s instructions. The membrane was as blocked with 5% skim milk in phosphate-buffered saline (PBS)-Tween (0.05% Tween-20 in PBS at pH 7.2, PBST) for 1 h at 37°C. The membrane was washed three times, and then was incubated with GST(91G1) rabbit mAb (1:1,000 dilution) at 4°C overnight. After washing three times with PBST, the membrane was incubated with HRP-labeled goat anti-rabbit IgG (1:3,000 dilution) at 37°C for 1 h. Then, the membrane was washed again and visualized using the ECL kit (Pierce). In addition, Western blot analysis was also performed using rabbit anti-CSBV IgG (1:1,000 dilution) as the first antibody and HRP-labeled goat anti-rabbit IgG (1:3,000 dilution) as the secondary antibody.

### Mice Immunizations

All animal procedures were approved by the Ethics Committee of Liaoning Medical University. Groups of four-to-six-week-old female pathogen-free BALB/c mice were immunized subcutaneously three times, at two-week intervals, with one of the three recombinant antigens (roVP1, roVP2, and roVP3) formulated in Freund’s adjuvant. Purified virus protein was used as a positive control, and PBS was used as a negative control. Additionally, a GST protein group was established. The amount of each antigen used for each immunization was 20 μg/mouse. Six mice from each group were bled, and serum samples were prepared prior to the first immunization and 2 weeks after each immunization, and then stored frozen until use.

### Detection of Mouse IgG Antibody by ELISA

Antigen-specific IgG of the serum samples was detected by ELISA [17]. Microtiter plates (Gibco, USA), were coated overnight at 4°C with 5 μg/mL (50 μL per well, 96-well plates) of the purified virus protein at 4°C overnight. The antigen-coated plates were washed three times with PBS-Tween, then blocked with 200 μL of blocking buffer (0.1% bovine serum albumin (BSA) for 1 h at 37°C. The blocked wells were washed three times with PBS. Samples that were to be tested for specific antibodies were diluted 1:500 with PBST. Plates were incubated at 37°C for 1 h, washed three times with PBST, and HRP-conjugated rabbit anti-mice IgG (1:3,000) was added to each well. The plates were incubated at 37°C for 1 h, and washed three times with PBST. After three washes, 100 μL of 3,3',5,5'-Tetramethylbenzidine(TMB) peroxidase substrate was added and incubated for 15 min at 37°C. Reactions were stopped by the addition of 2 M HCl, and the absorbance was read at 450 nm (OD_450_) on an ELISA plate reader.

### Lymphocyte Proliferation Assay

Lymphocyte proliferation of the immunized rats was detected by the MTT assay. Six mice from the different groups were sacrificed by cervical dislocation two weeks after the last immunization, and splenocyte suspensions were separated as previously described [18]. Splenocytes were seeded into 96-well culture plates at a density of 2.0×10^6^ cells/well in RPMI 1640 medium containing 10% fetal bovine serum (Invitrogen). The cells were stimulated with 20 μg/mL of purified virus protein for 72 h at 37°C under 5% CO_2_, followed by the addition of 10 μL/well of MMT (10 μg/mL). Then, the plates were incubated for 4 h at 37°C, and 150 μL/well of dimethyl sulfoxide (DMSO) solution was added to the culture plates. At the same time, medium alone was used as a negative control. Finally, the absorbance values of the samples were measured at 570 nm. The lymphocyte proliferation rate was quantified by the stimulation index (SI), which was calculated as the ratio of the OD_570_ of the stimulated cells to the OD_570_ of the negative controls.

### Statistical Analysis

A one-way analysis of variance (ANOVA) test with Tukey post-hoc test in GraphPad Prism version 5.0 was used in this study, and the data were expressed as mean ± standard deviation (SD). Values of p<0.05 were considered to be statistically significant.

## Results

### Construction of Wild Type Gene Recombinant Plasmids

**Fig 1:**
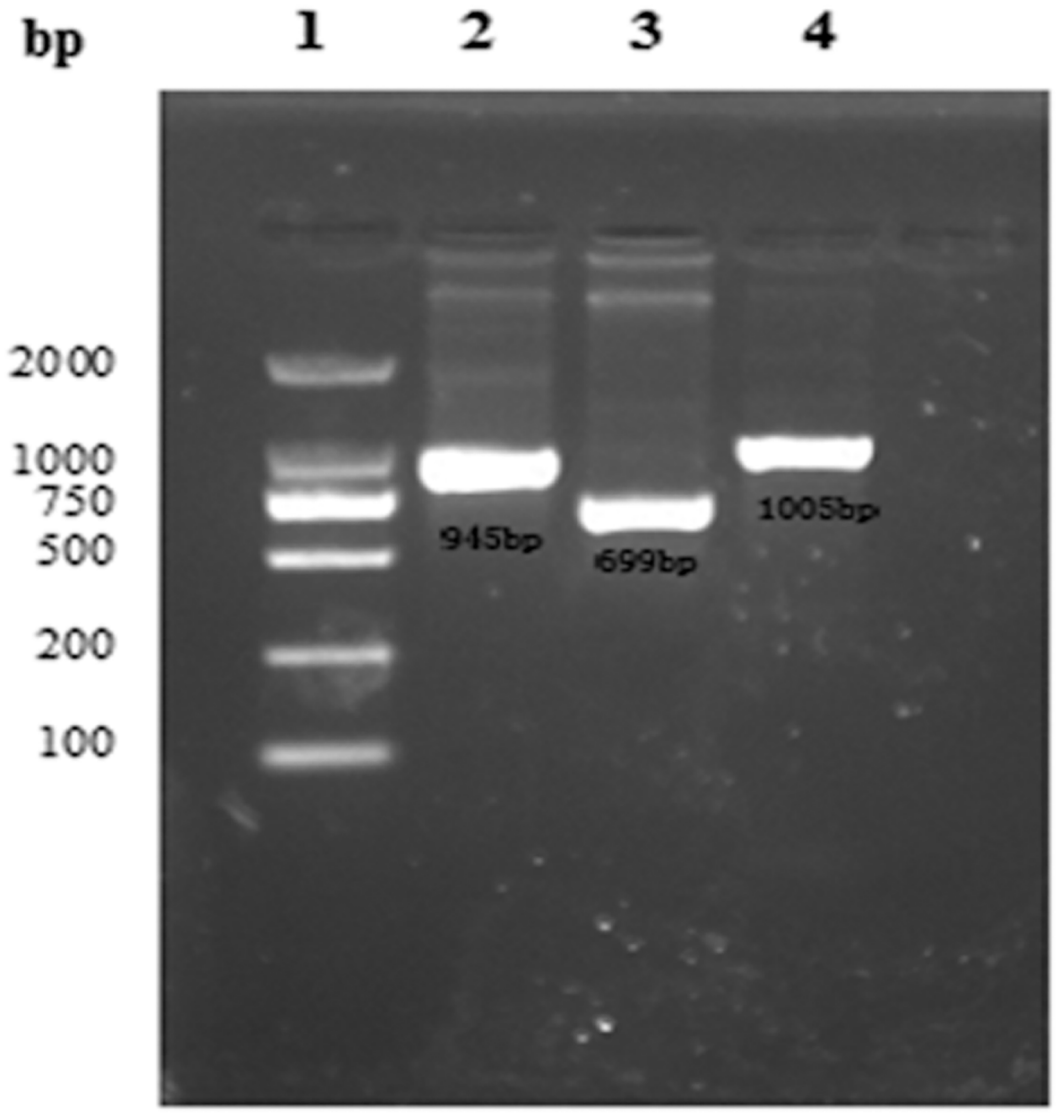
PCR results for the CSBV *VP1*, *VP2*, and *VP3* genes. Lane 1, DNA marker DL2000; Lane 2, *VP1* gene PCR products; Lane 3, *VP2* gene PCR products; Lane 4, *VP3* gene PCR products.

The structural genes *VP1*, *VP2*, and *VP3* were amplified using the primers shown in Table 1, and the results showed that fragment lengths of about 945, 699, and 1,005 bp were obtained by PCR amplification, which were the same as expected (Fig 1). After the gene fragments were inserted into the pGEX-6P-1 vector, restriction digestion analysis and sequencing showed that the non-optimized gene expression vectors were successfully constructed.

### Recoding of the *VP1*, *VP2*, and *VP3* Genes, and Identification of Recoding Recombinant Plasmids

The Genbank sequence (accession no. HM237361.1) was used as a reference sequence for the synthesized genes; 260 of 945 nt, 178 of 699 nt, and 253 of 1,005 nt of the *VP1*, *VP2*, and *VP3* genes, respectively, were substituted. Codons in the wild-type *VP1*, *VP2*, and *VP3* genes were replaced by the preferred codons of *E*. *coli*, and the codon distribution of *oVP1*, *oVP2*, *oVP3* and wild type genes are shown in Table 2. In the reference sequence, runs of five or more nucleotides were eliminated after recoding. The number of homopolymeric sequences containing more than five nucleotides in *VP1*, *VP2* and *VP3* genes were four, one and three, respectively. After recoding, codon adaptation index (CAI) of *oVP1*, *oVP2*, and *oVP3* genes reached to 0.957, 0.952 and 0.966 using online optimization software (http://www.jcat.de/ and http://genomes.urv.es/OPTIMIZER/), respectively, while the substitution of nucleotides did not alter the amino acid-encoding sequences (S1 Fig). The recombinant plasmids were successfully confirmed by double-digestion identification and sequencing.

**Table 2:** Codon distribution of *oVP1*, *oVP2*, *oVP3* and wild type sequences.

Codon	Wild type			Recoding			Codon	Wild type			Recoding			Codon	Wild type			Recoding			Codon	Wild type			Recoding		
	*VP1*	*VP2*	*VP3*	*oVP1*	*oVP2*	*oVP3*		*VP1*	*VP2*	*VP3*	*oVP1*	*oVP2*	*oVP3*		*VP1*	*VP2*	*VP3*	*oVP1*	*oVP2*	*oVP3*		*VP1*	*VP2*	*VP3*	*oVP1*	*oVP2*	*oVP3*
**GCA (A)**	**5**	**3**	**7**	**0**	**0**	**0**	**GCC (A)**	**2**	**2**	**2**	**0**	**0**	**0**	**GCG (A)**	**6**	**3**	**2**	**18**	**15**	**19**	**GCT (A)**	**5**	**7**	**8**	**0**	**0**	**0**
**TGC (C)**	**0**	**0**	**1**	**1**	**2**	**4**	**TGT (C)**	**1**	**2**	**3**	**0**	**0**	**0**	**GAC (D)**	**4**	**1**	**5**	**21**	**9**	**21**	**GAT (D)**	**17**	**8**	**16**	**0**	**0**	**0**
**GAA (E)**	**3**	**8**	**10**	**11**	**11**	**18**	**GAG (E)**	**8**	**3**	**8**	**0**	**0**	**0**	**TTC (F)**	**4**	**2**	**1**	**13**	**13**	**16**	**TTT (F)**	**9**	**11**	**15**	**0**	**0**	**0**
**GGA (G)**	**8**	**5**	**7**	**0**	**0**	**0**	**GGC (G)**	**0**	**1**	**1**	**0**	**0**	**0**	**GGG (G)**	**3**	**4**	**6**	**0**	**0**	**0**	**GGT (G)**	**7**	**3**	**10**	**18**	**13**	**24**
**CAC (H)**	**4**	**2**	**4**	**8**	**4**	**8**	**CAT (H)**	**4**	**2**	**4**	**0**	**0**	**0**	**ATA (I)**	**11**	**8**	**8**	**0**	**0**	**0**	**ATC (I)**	**0**	**1**	**1**	**13**	**16**	**16**
**ATT (I)**	**2**	**7**	**7**	**0**	**0**	**0**	**AAA (K)**	**8**	**7**	**6**	**15**	**13**	**10**	**AAG (K)**	**7**	**6**	**4**	**0**	**0**	**0**	**TTA (L)**	**6**	**5**	**6**	**0**	**0**	**0**
**TTG (L)**	**11**	**4**	**8**	**0**	**0**	**0**	**CTA (L)**	**0**	**0**	**0**	**0**	**0**	**0**	**CTC (L)**	**0**	**0**	**0**	**0**	**0**	**0**	**CTG (L)**	**0**	**3**	**2**	**17**	**13**	**18**
**CTT (L)**	**0**	**1**	**2**	**0**	**0**	**0**	**ATG (M)**	**5**	**6**	**14**	**5**	**6**	**14**	**AAC (N)**	**1**	**1**	**6**	**17**	**15**	**19**	**AAT (N)**	**16**	**14**	**13**	**0**	**0**	**0**
**CCA (P)**	**6**	**6**	**4**	**0**	**0**	**0**	**CCC (P)**	**1**	**0**	**3**	**0**	**0**	**0**	**CCG (P)**	**8**	**3**	**5**	**22**	**12**	**19**	**CCT (P)**	**7**	**3**	**7**	**0**	**0**	**0**
**CAA (Q)**	**7**	**8**	**4**	**0**	**0**	**0**	**CAG (Q)**	**2**	**8**	**8**	**9**	**16**	**12**	**AGA (R)**	**10**	**2**	**6**	**0**	**0**	**0**	**AGG (R)**	**2**	**3**	**1**	**0**	**0**	**0**
**CGA (R)**	**3**	**4**	**0**	**0**	**0**	**0**	**CGC (R)**	**1**	**1**	**1**	**0**	**0**	**0**	**CGG (R)**	**1**	**1**	**4**	**0**	**0**	**0**	**CGT (R)**	**5**	**2**	**3**	**22**	**13**	**15**
**AGC (S)**	**2**	**0**	**0**	**0**	**0**	**0**	**AGT (S)**	**9**	**4**	**7**	**0**	**0**	**0**	**TCA (S)**	**10**	**3**	**4**	**0**	**0**	**0**	**TCC (S)**	**0**	**1**	**2**	**0**	**0**	**0**
**TCG (S)**	**1**	**4**	**1**	**0**	**0**	**0**	**TCT (S)**	**7**	**3**	**6**	**29**	**15**	**20**	**ACA (T)**	**4**	**8**	**6**	**0**	**0**	**0**	**ACC (T)**	**1**	**2**	**3**	**24**	**19**	**30**
**ACG (T)**	**5**	**6**	**6**	**0**	**0**	**0**	**ACT (T)**	**14**	**3**	**15**	**0**	**0**	**0**	**GTA (V)**	**9**	**5**	**15**	**0**	**0**	**0**	**GTC (V)**	**0**	**0**	**1**	**0**	**0**	**0**
**GTG (V)**	**9**	**3**	**8**	**0**	**0**	**0**	**GTT (V)**	**12**	**7**	**7**	**30**	**15**	**31**	**TGG (W)**	**5**	**1**	**5**	**5**	**1**	**5**	**TAC (Y)**	**3**	**6**	**3**	**17**	**12**	**16**
**TAT (Y)**	**14**	**6**	**13**	**0**	**0**	**0**	**TAA (.)**	**0**	**0**	**0**	**0**	**0**	**0**	**TGA (.)**	**0**	**0**	**0**	**0**	**0**	**0**	**TAG (.)**	**0**	**0**	**0**	**0**	**0**	**0**

### Expression and Purification of the Wild Type and Recoding Gene Plasmids

**Fig 2:**
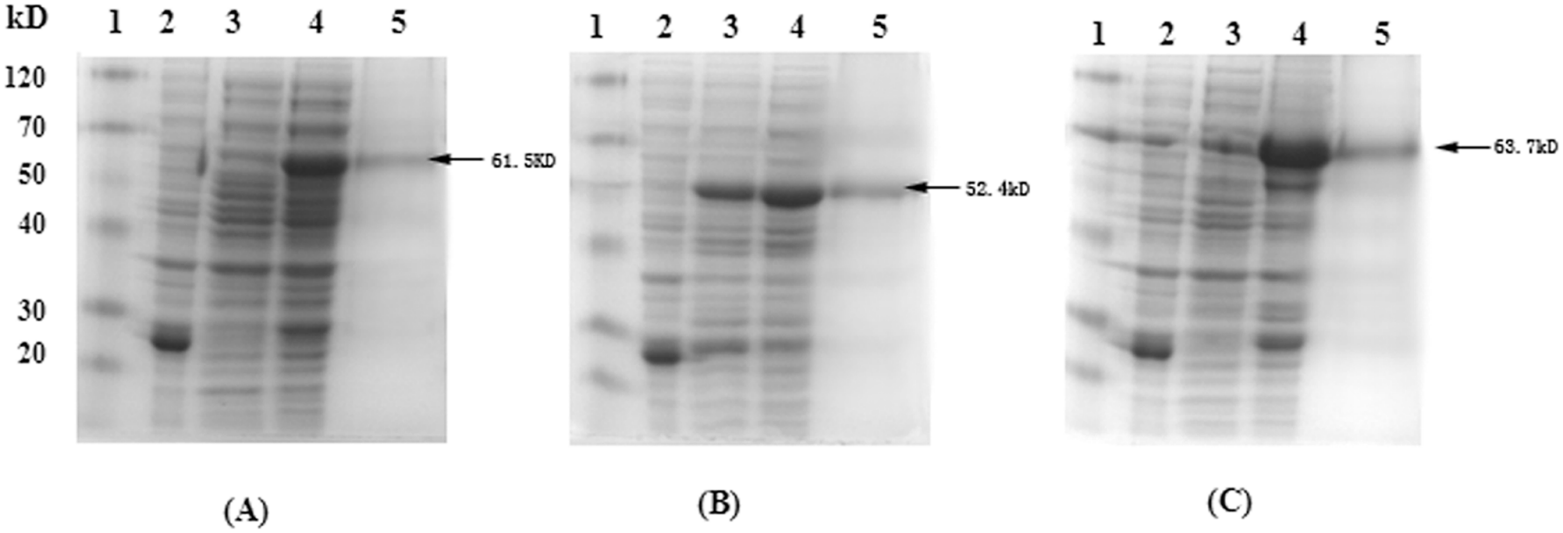
Expression and purification of recombinant proteins. A, B, and C represent roVP1, roVP2, and roVP3 proteins, respectively. SDS-PAGE analysis of roVP1, roVP2, and roVP3 proteins were as follows: Lane 1, low molecular weight protein marker; Lane 2, pGEX-6P-1 vector after induction; Lane 3, wild type gene expression after induction; Lane 4, recoding gene expression after induction; Lane 5, purification of expressed proteins using GST-agarose affinity columns.

The expression plasmids were transformed into BL21(DE3). After induction, protein expression levels were analyzed by SDS-PAGE. The results indicated that the wild type genes were not expressed or expressed at low levels, while the recoding genes were highly expressed in the culture supernatant, and the molecular weights of the GST-tagged recombinant proteins were about 61.5 kDa (roVP1), 52.4 kDa (roVP2), and 63.7 kDa (roVP3) (Fig 2). The results were in accordance with the theoretical values. The best induction conditions of the engineered genes were: *oVP1* gene, 0.2 mmol/L IPTG and 5 h at 30°C; *oVP2* gene, 0.25 mmol/L IPTG and 5 h at 25°C; *oVP3* gene, 0.2 mmol/L IPTG and 5 h at 30°C. Each protein could be purified effectively using the GST-Tagged Protein Purification Kit (Fig 2), and the overall yields of roVP1, roVP2, and roVP3 proteins were around 1.68, 1.90, and 2.26 mg per gram of pelleted bacteria, respectively, as assessed by the BCA assay.

### Western Blot Analysis of Recombinant Proteins

**Fig 3:**
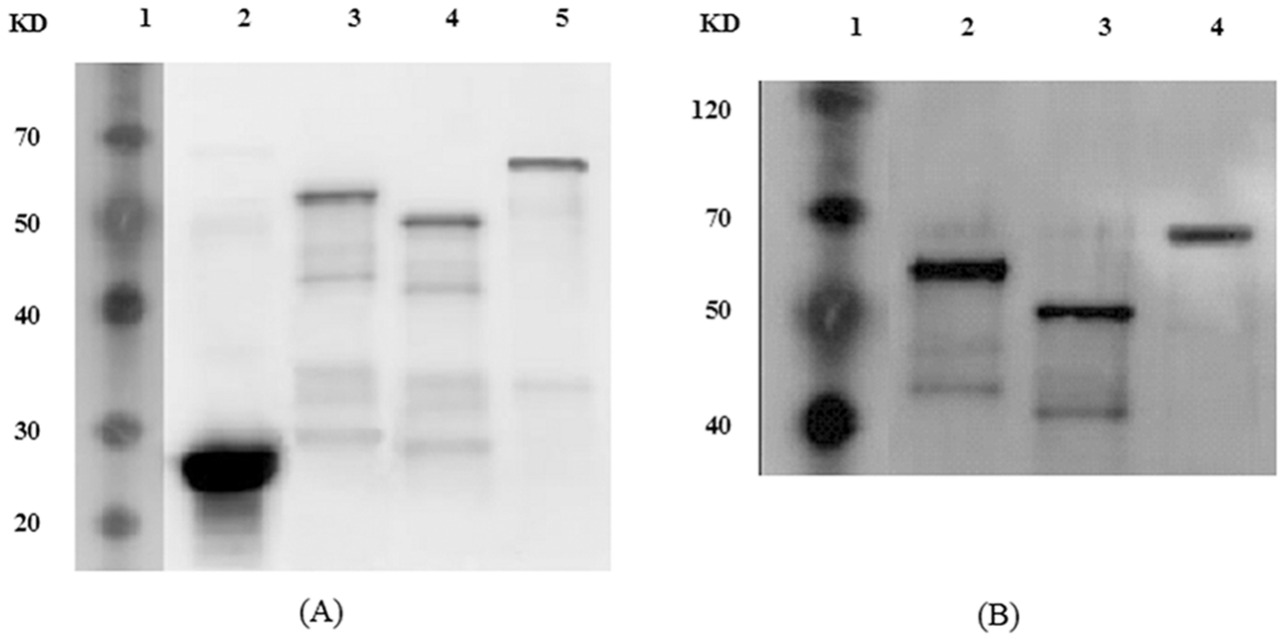
Western blot analysis of recombinant proteins. A (anti-GST rabbit mAb as primary antibody): Lane 1, low molecular weight protein marker; Lane 2, GST protein; Lane 3, roVP1 protein; Lane 4, roVP2 protein; Lane 5, roVP3 protein. B (rabbit anti-CSBV IgG as primary antibody): Lane 1, low molecular weight protein marker; Lane 2, roVP1 protein; Lane 3, roVP2 protein; Lane 4, roVP3 protein.

Protein expression was detected by western bolt analysis using GST rabbit mAb or rabbit anti-CSBV IgG as the first antibody. The results showed that the recombinant proteins reacted specifically with the first antibody, and had molecular weights of 61.5, 52.4, and 63.7 kDa for roVP1, roVP2, and roVP3, respectively. In the negative control, however, the GST protein appeared at about 26 kDa (Fig 3). The results were in accordance with the SDS-PAGE analysis, and the molecular weights were consistent with the calculated values.

### Differential Response of Specific IgG during the Immunization

**Fig 4:**
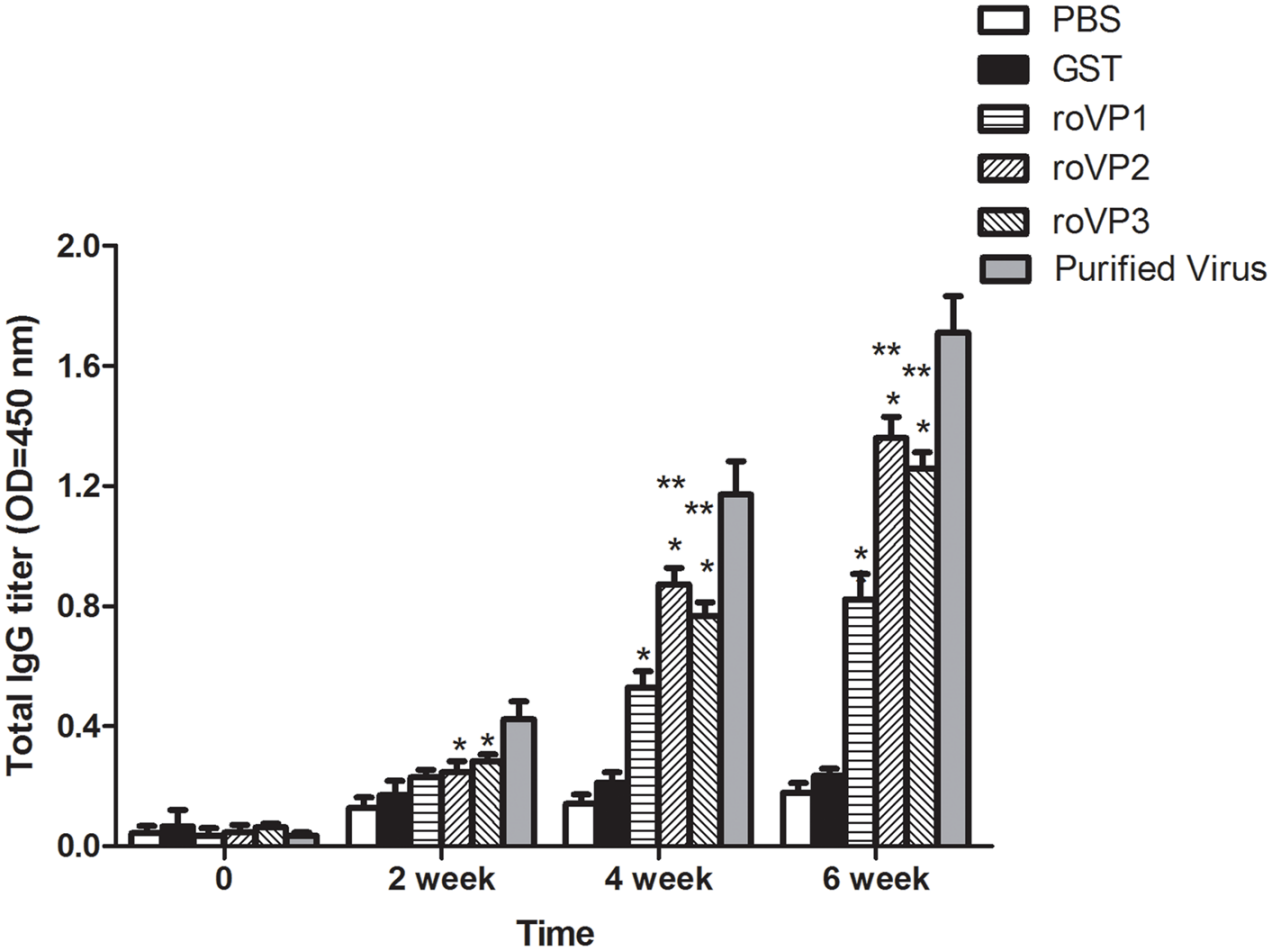
Specific IgG antibodies in serum of the immunized mice. *Significantly different than the GST-immunized group (p<0.05). **Significant differences between the three groups immunized with recombinant proteins (p<0.05).

To assess the immunogenicity of the recombinant proteins, the mice were immunized with roVP1, roVP2, and roVP3 in combination with Freund’s adjuvant, and the IgG levels specific to the different antigens are shown in Fig 4 (S1 Table). In the test, We found no significant difference between PBS group and GST group (p>0.05). As expected, the vaccination of mice with purified virus protein induced significantly higher levels of specific antibodies than those of other groups. In the groups immunized with recombinant proteins, the roVP2 group presented the highest levels of antibodies after the booster immunization, the roVP3 group had the second highest, and the roVP1 group produced the lowest immune response; the differences between the roVP2 and roVP3 groups was significant (p<0.05). The GST group did not react with the antigen. After booster immunizations, the three groups immunized with the recombinant proteins exhibited significantly higher immune responses than the GST group (p<0.05).

### Lymphocyte Proliferation

To evaluate the lymphocyte proliferation of the mice in the immunized groups, we re-stimulated the lymphocytes with purified virus protein in vitro, and observed increases in their proliferation (S2 Table). After re-stimulation with the antigen, the lymphoproliferation of mice immunized with purified virus protein was the most significant, and the SI value (SI = 1.842±0.063) was the largest of all the groups (Table 3). Splenic lymphocytes of the three groups immunized with the recombinant proteins exhibited different proliferation effects, and the SI values of the roVP1(SI = 1.573±0.040), roVP2(SI = 1.675±0.059), and roVP3(SI = 1.628±0.075) groups were significantly different than the GST group (p<0.05), although the SI values of the recombinant protein groups were not significantly (Table 3). The SI values of the GST (SI = 1.122±0.056) and control groups (SI = 1.088±0.039) were not significantly different.

**Table 3:** Lymphocyte proliferation responses in different immunized and control groups.

Group	Mean Stimulation Index (SI)
PBS	1.088±0.039^c^
GST	1.122±0.056^c^
roVP1	1.573±0.040^b^
roVP2	1.675±0.059^b^
roVP3	1.628±0.075^b^
Purified Virus	1.842±0.063^a^

Different superscript letters indicate that the differerces were significant (p<0.05), same superscript letters indicate that the differerces were not significant (p>0.05).

## Discussion

CSBV infections in *A*. *cerana* have endangered not only the honeybee industry, but also represent a potential threat to the agricultural production of crops dependent on pollination [19,20]. Currently, CSBV research is focused on genetic characterization, rapid detection, virus culture systems, and treatment [21–24]. As we know that structural proteins play an important role in viral infection, replication, pathogenicity, etc. [25–27], the study of CSBV protein is not sufficient, especially for structural proteins. In our study, we have successfully produced roVP1 protein, roVP2 protein, and roVP3 protein using a prokaryotic expression system to evaluate their immunogenicity, and Western blot analysis of the recombinant fusion proteins confirmed that they were specifically recognized by anti-GST and rabbit anti-CSBV antibodies. These assays indicated that the structural protein genes were expressed at high levels in *E*. *coli*.

The advantages of using pGEX-6P-1 as the expression vector in this study was as follows [28]: first, the expressed fusion proteins were soluble, thereby avoiding the renaturation process that is required for proteins that form inclusion bodies; second, pGEX-6P-1 features a GST tag, and the tag permits the purification of the expressed protein by a GST-agarose affinity column, and the GST tag can be cleaved from the recombinant fusion protein using PreScission protease, thrombin, or coagulation factor Xa. All of these advantages provide favorable conditions for the further research of CSBV.

Generally, there are significant differences in codon preference between different organisms, as well as between different species. Thus, codon optimization has been used to increase protein expression [29–31]. In previous research, we inserted the wild-type sequences of CSBV *VP1*, *VP2*, and *VP3* into the pGEX-6P-1 vector, although their expression levels were very low. In the present study, the strategy of codon optimization was utilized without modifying the amino acid sequences of the encoded proteins, which was accomplished by the “one-codon” approach. The results revealed that the codon recoding of *VP1*, *VP2*, and *VP3* genes was successful.

In our previous study, we had found that immunoglobulin Y (IgY) against CSBV by purified virus could effectively treat colonies infected with CSBV, and this result show that the heterogenous antibody work against CSBV infection, but there isn’t a method to incubate virus at present. So the purpose for assessing the immunogenicity of recombinant protein is to prepare polyclonal antibodies, and this work laid the foundation for developing new diagnostic kits, and further functional studies of the CSBV structural proteins. At the same time, polyclonal antibodies could be used as preventive and therapeutic treatments for CSBV infections, and the research provides some new ideas regarding viral diseases of honeybees. In this study, the IgG levels and SI values of splenic lymphocyte proliferation were selected as the indicators of immunogenicity.

Honeybee immune pathways have large differences in comparison with vertebrate immune systems. The honeybee immune pathway lacks adaptive immunity [32], but shows many parallels with the innate immune response of vertebrates, mainly involving a series of actions, including the secretion of antimicrobial peptides, phagocytosis, melanization, and the enzymatic degradation of pathogens [33–35]. Thus, the indices of IgG subtypes and T cells subsets have little significance to the honeybee. After evaluating their antigenicity in mice, the roVP2 and roVP3 proteins proved to have better immunogenicity than the roVP1 protein. Thus, roVP2 and roVP3 were thought to be good candidates as immunogens for polyclonal antibody production and diagnostic methods. In the lymphocyte proliferation assay, the purified virus protein stimulated splenocyte growth effectively, and the SI values of the three recombinant protein groups were not significantly different, but there were significant difference between the recombinant protein groups and the GST group (p<0.05). To avoid interference of GST protein, the purified virus protein was used as the re-stimulated antigen. The lymphocyte proliferation assay results further verified that the roVP2 and roVP3 proteins possessed the immunogenicity needed to prepare polyclonal antibodies, and that the roVP1 protein might be relevant to cellular immunity. Because of the specificity of the honeybee immune mechanism, the effect of recombinant proteins on the honeybee immune system requires further study.

In summary, in this study, the structural protein genes *VP1*, *VP2*, and *VP3* of CSBV were recoded and successfully expressed in *E*. *coli* for the first time. The results of this study proved that the recombinant proteins roVP2 and roVP3 were highly immunogenic in mice, and are good candidate immunogens for polyclonal antibody production and further research.

## Supporting Information

S1 Fig The result of recoding *VP1*, *2*, and *3* genes. (DOC)

S1 Table The data of specific IgG antibodies in serum of the immunized mice. (XLS)

S2 Table The data of SI values in the Lymphocyte proliferation. (XLS)
