# Correction: Codon Optimization, Expression in *Escherichia coli*, and Immunogenicity of Recombinant Chinese Sacbrood Virus (CSBV) Structural Proteins VP1, VP2, and VP3

**DOI:** 10.1371/journal.pone.0134423

**Published:** 2015-07-28

**Authors:** Dongliang Fei, Haochun Zhang, Qingyun Diao, Lili Jiang, Qiang Wang, Yi Zhong, Zhaobin Fan, Mingxiao Ma

The following information is missing from the Funding section: This study was supported by the Agricultural Science and Technology Innovation Program (No. CAAS-ASTIP-2015-IAR).
