# Supplementary material for: Codon Optimization, Expression in Escherichia coli, and Immunogenicity of Recombinant Chinese Sacbrood Virus (CSBV) Structural Proteins VP1, VP2, and VP3
Source: PLoS One. 2015 Jun 12;10(6):e0128486. doi: 10.1371/journal.pone.0128486 (PMC4466328; doi:10.1371/journal.pone.0128486)

Figure 1. Sequences of non-optimized and optimized VP1 gene by online optimization software (http://genomes.urv.es/OPTIMIZER/)


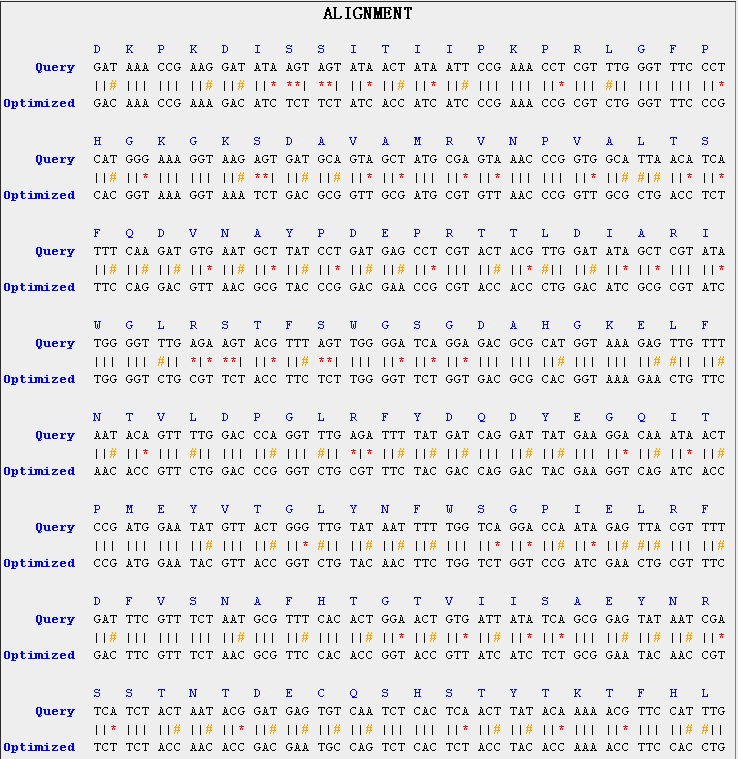


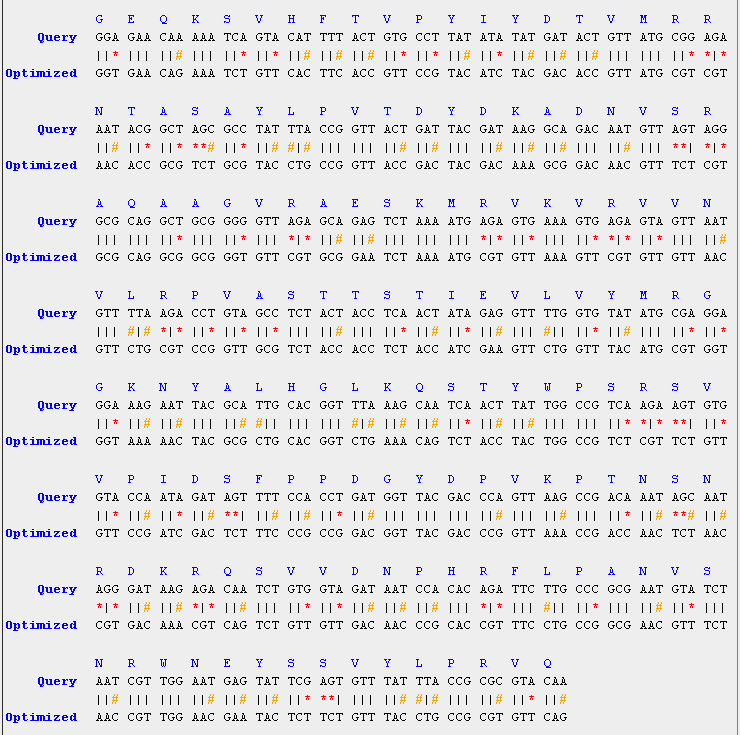


**| : Unchanged nucleotide 
    * : Transversion change (Purines <-> Pyrimidines) 
    # : Transition change (Purine <-> Purine / Pyrimidine <-> Pyrimidine)**

Figure 2. Sequences of non-optimized and optimized VP2 gene by online optimization software(http://genomes.urv.es/OPTIMIZER/)


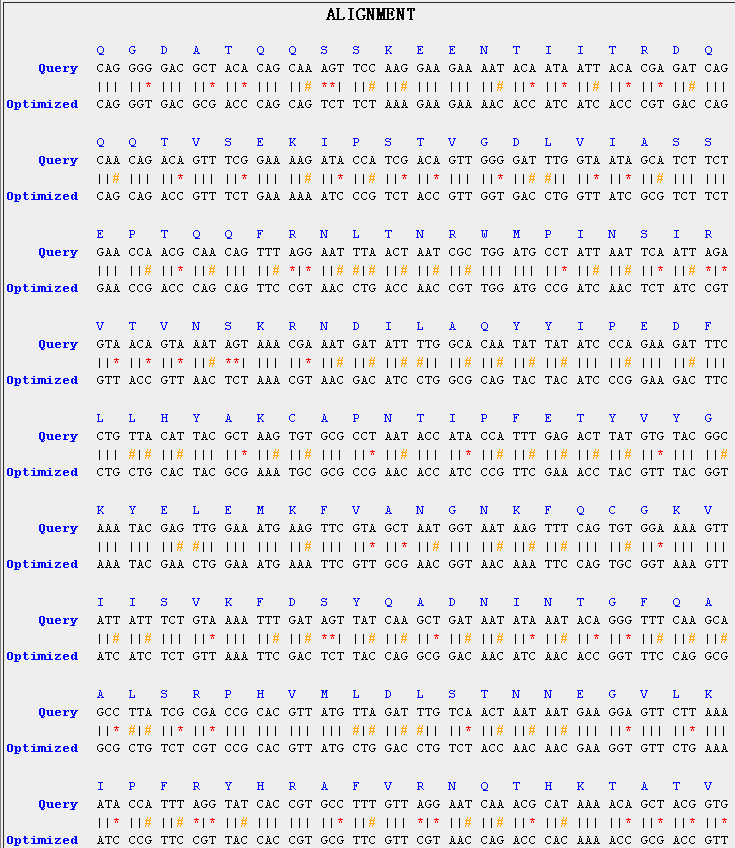

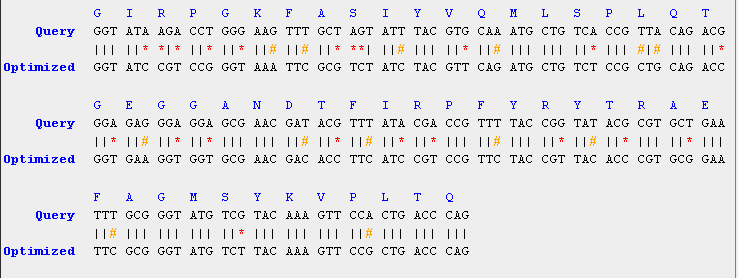


**| : Unchanged nucleotide 
    * : Transversion change (Purines <-> Pyrimidines) 
    # : Transition change (Purine <-> Purine / Pyrimidine <-> Pyrimidine)**

Figure 3. Sequences of non-optimized and optimized VP3 gene by online optimization software(http://genomes.urv.es/OPTIMIZER/)


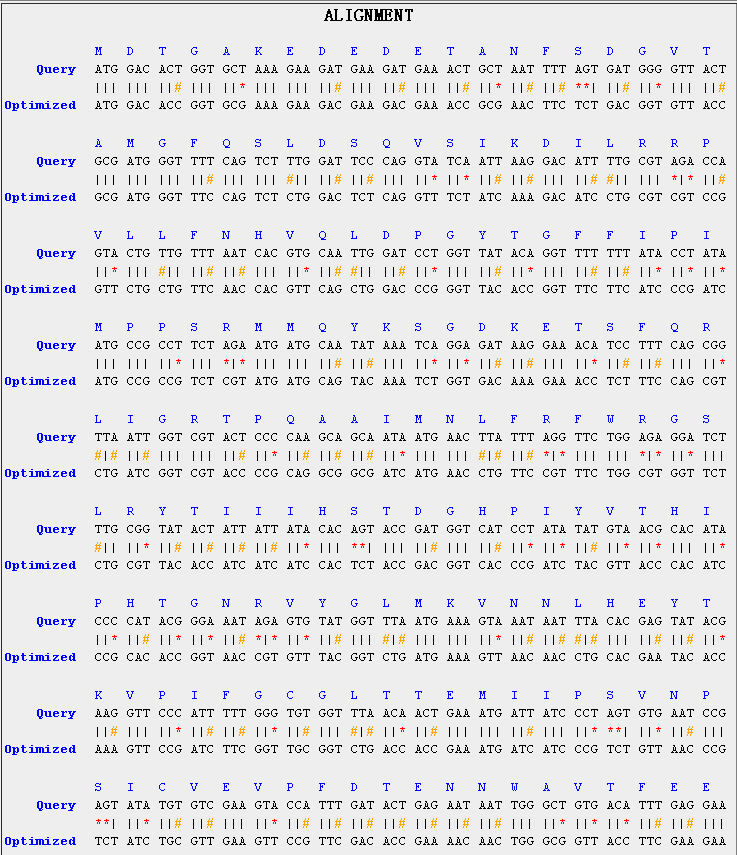

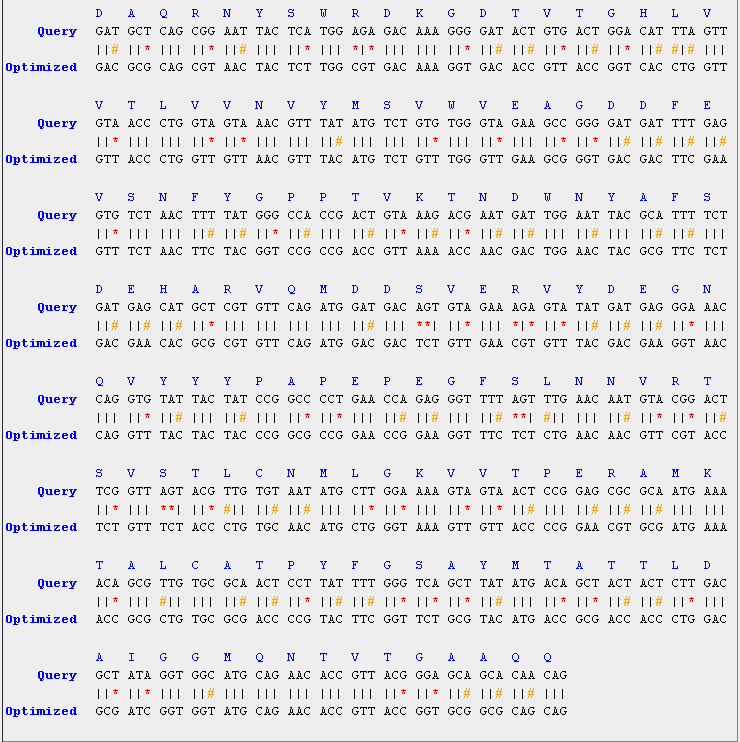


**| : Unchanged nucleotide 
    * : Transversion change (Purines <-> Pyrimidines) 
    # : Transition change (Purine <-> Purine / Pyrimidine <-> Pyrimidine)**

Figure 4. Codon adaptation index (CAI) before recoded and after recoded of VP1 gene by online optimization software (http://www.jcat.de/)

Before recoded


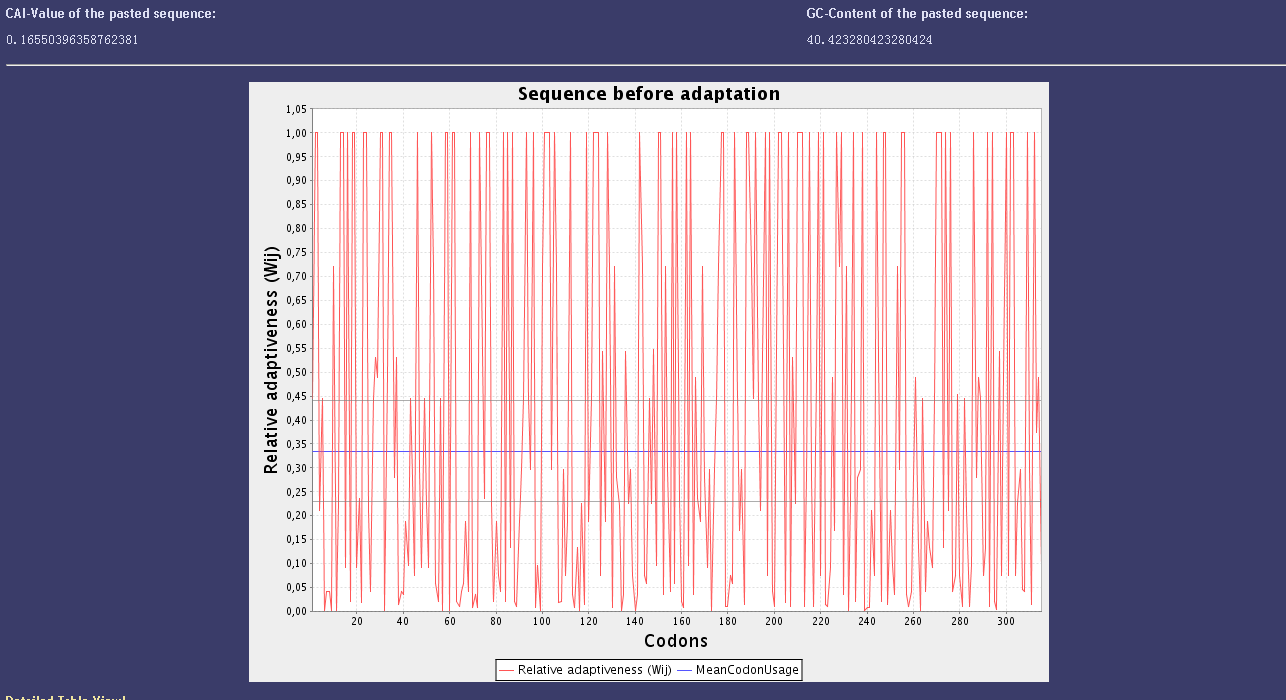


After recoded


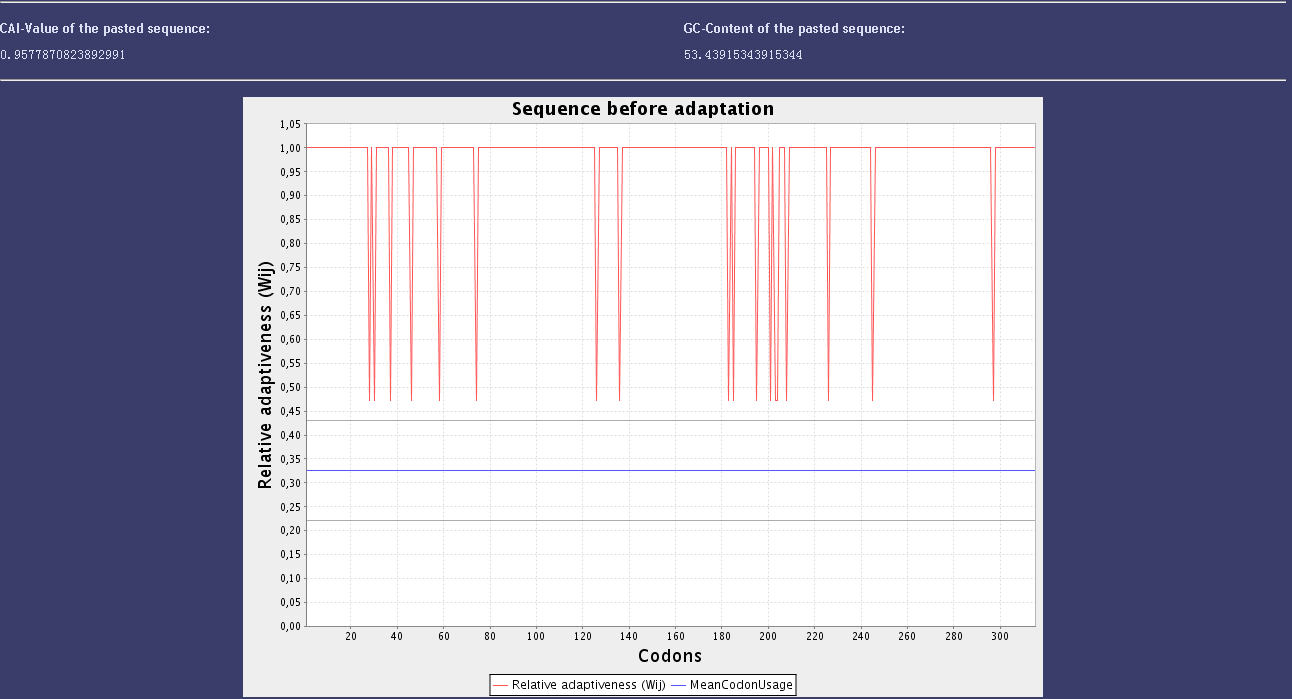


Figure 5. Codon adaptation index (CAI) before recoded and after recoded of VP2 gene by online optimization software(http://www.jcat.de/)

Before recoded


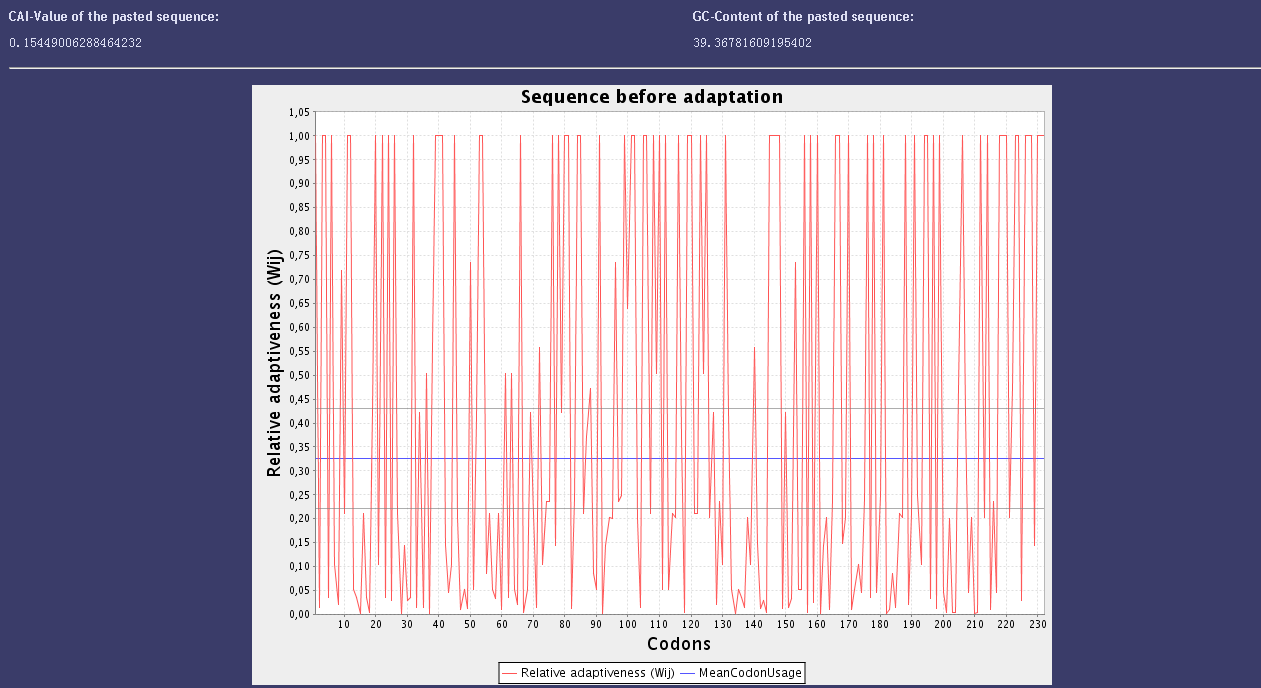


After recoded


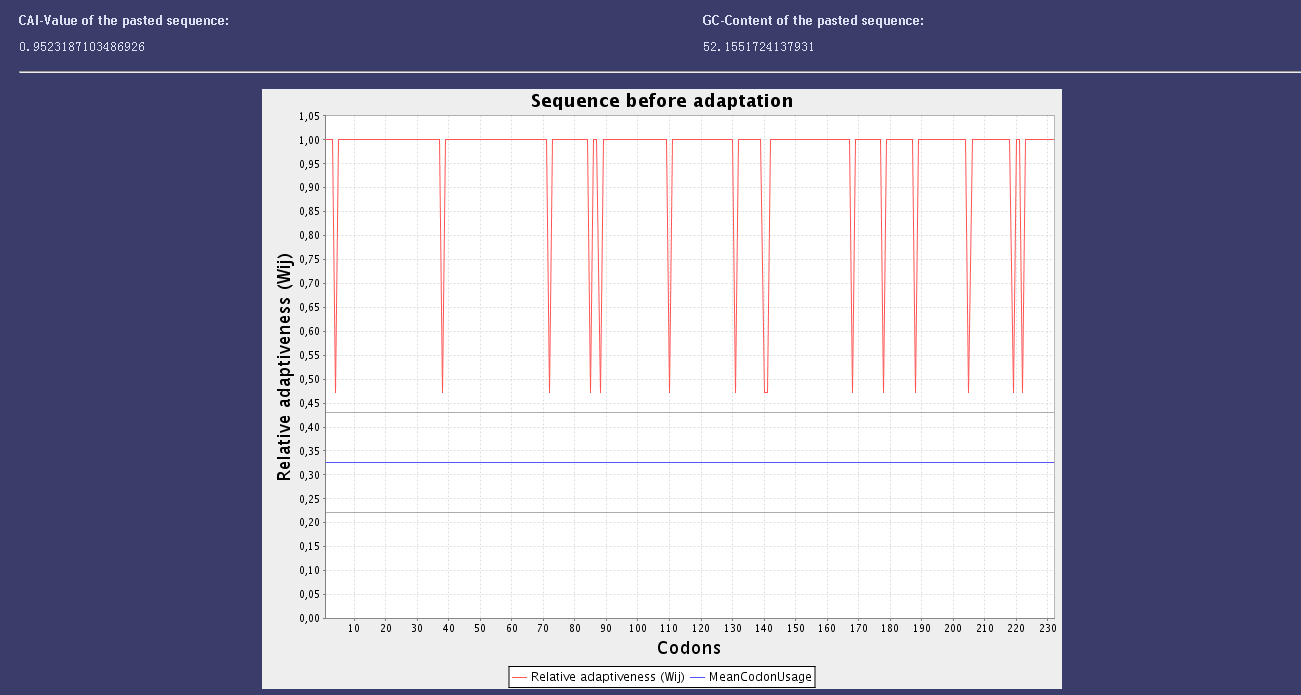


Figure 6. Codon adaptation index (CAI) before recoded and after recoded of VP3 gene by online optimization software(http://www.jcat.de/)

Before recoded


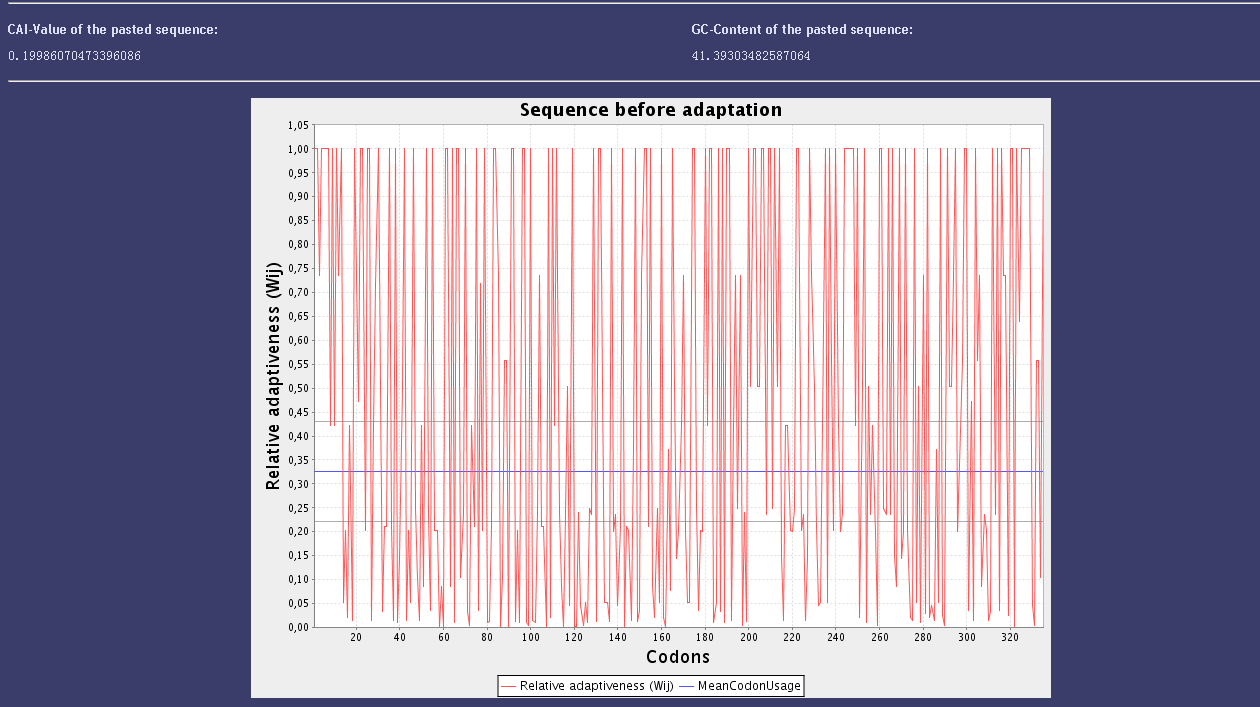


After recoded


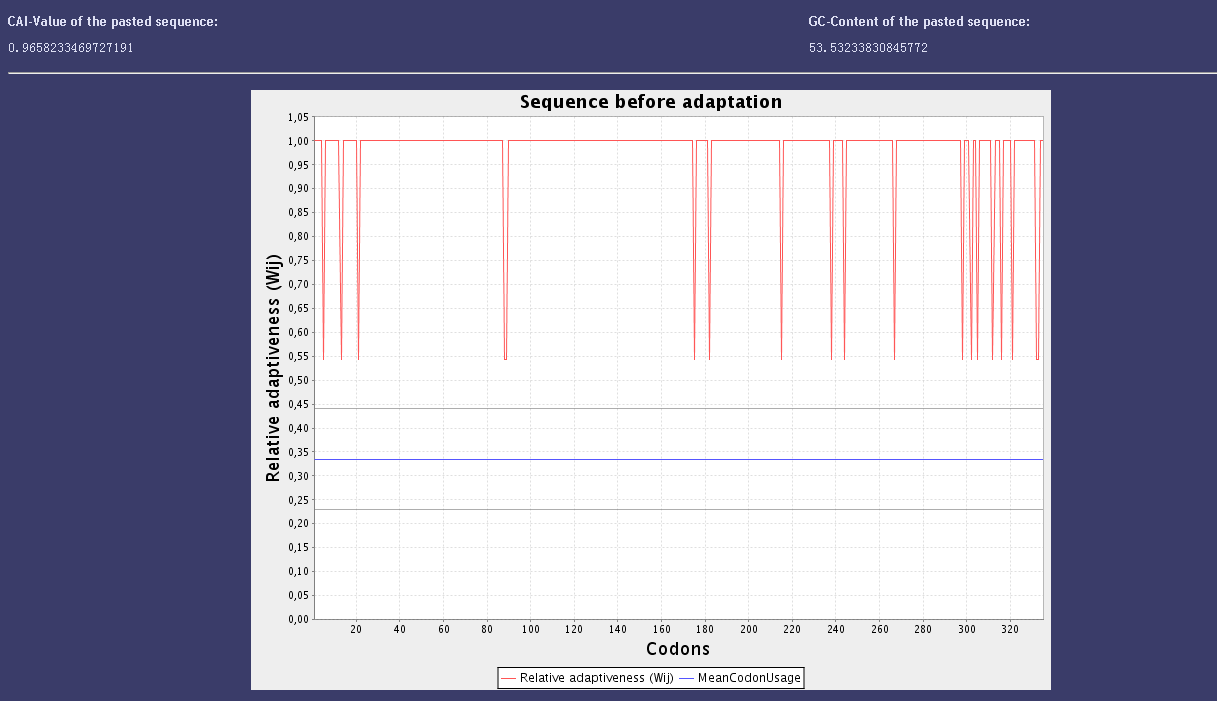

Supplement: S1 Fig — (DOC) [file pone.0128486.s001.doc]
